# 87 Comparing the Efficiency of Tumescent Infiltration Techniques in Burn Surgery

**DOI:** 10.1093/jbcr/irac012.090

**Published:** 2022-03-23

**Authors:** Tom Fouché, Stephanie Bond, Sebastian Q Vrouwe

**Affiliations:** University of Chicago Pritzker School of Medicine, Chicago, Illinois; University of Chicago, Chicago, Illinois; University of Chicago, Chicago, Illinois

## Abstract

**Introduction:**

Tangential excision and grafting of burn wounds results in significant intra-operative blood loss. Infiltration of a dilute epinephrine solution is routinely performed to reduce bleeding from both the eschar and donor sites. Tumescence infiltration has been described using both a manual technique or a pump device. The purpose of this study is to compare the efficiency of these two methods in terms of time and economy of motion.

**Methods:**

Consecutive adult burn patients who required excision and grafting were enrolled in the study. Manual infiltration consisted of an aspirating syringe (10 mL), spinal needle (18 gauge), and three-way stopcock connected to sterile tubing. Pump infiltration consisted of a blunt cannula and infiltration pump (300 mL/minute). Prior to each case, patients were randomized into either the manual or pump tumescent technique. Excisions of < 2% TBSA were excluded, along with specific anatomic regions (head and neck, genitalia, hands and forearms, feet, and legs below the knee). Infiltration with epinephrine-saline solution (1:500,000) was performed to the endpoint of tissue tumescence by a single surgeon for consistency. ImageJ software was used to calculate the surface area to be infiltrated. The infiltration was filmed and later coded for duration, number of maneuvers, and total volume of tumescence injected. Any complications related to the infiltration of tumescence were collected.

**Results:**

A total of 14 patients were enrolled, and 16 cases were randomized to either manual (N=8) or pump infiltration (N=8). Participants were 71% male, with a mean age of 41.8 years, and mean burn size of 16.9% TBSA. The pump method required less time (2.0 cm^2^/s vs 1.1 cm^2^/s, p< 0.001) and fewer maneuvers (37.8 cm^2^/move vs 1.1 cm^2^/move, p< 0.001). Use of the infiltration pump also resulted in a reduced volume of fluid required to reach the endpoint of tumescence (1.7 mL/cm^2^ vs 2.4 mL/cm^2^, p=0.01). No complications were reported related to the process of tumescence in either group.

**Conclusions:**

Compared to the manual technique, use an infiltration pump was significantly more efficacious in terms of both time and economy of motion. Additionally, less fluid volume was required to achieve the endpoint of tumescence.

